# Telehealth for Addressing Climate Change: A Position Statement

**DOI:** 10.1055/s-0046-1816597

**Published:** 2026-11-03

**Authors:** Anandhi Ramachandran, Shashi Gogia, Gumindu Kulatunga, Craig Kuziemsky, Sheila John, Tania Moerenhout, Marcia Ito, Prasad Ranatunga, Usman Iqbal, Kleber Arajuo, Ho Kendall, Inga Hunter, Sriram Iyengar, Arindam Basu

**Affiliations:** 1 International Institute of Health Management ResearchDelhiIndia; 2 Society for Administration of Telemedicine and Healthcare InformaticsNew DelhiIndia; 3 Health Information Unit, Ministry of HealthColomboSri Lanka; 4 Office of Research Services, MacEwan UniversityEdmontonAlbertaCanada; 5 Medical Research Foundation, Sankara NethralayaChennaiIndia; 6 Bioethics Centre, University of Otago (Otakou Whakaihu Waka)AotearoaNew Zealand; 7 Paula Souza State Center for Technological EducationSão PauloBrazil; 8 Provincial Department of Health Services - North-Western ProvinceSri Lanka; 9 School of Population Health, Faculty of Medicine and Health, University of New South WalesSydneyAustralia; 10 Medicina Interna e Saúde DigitalRecifePernambucoBrazil; 11 Department of Emergency Medicine, The University of British ColombiaCanada; 12 School of Management, Massey UniversityNew Zealand; 13 University of Arizona College of MedicinePhoenixUSA; 14 School of Health Sciences, University of CanterburyNew Zealand

**Keywords:** Climate Change, Telehealth, Greenhouse Gas, e-waste, Delivery of Healthcare, Healthcare, Systems Thinking, Position Statement

## Abstract

**Introduction:**

The health sector is a major contributor to climate change through greenhouse gas emissions. Telehealth, the delivery of healthcare services remotely through communication technologies is on the rise globally as evidenced during the COVID-19 pandemic. Here we express our opinion through a position statement of how telehealth can be effectively adopted for addressing climate change mitigation using a systems thinking approach.

**Methods:**

We conducted a narrative review of the literature and an online modified Delphi method among the International Medical Informatics Association (IMIA) Telehealth Working Group members to develop a set of themes for an evidenced based position statement on the adoption of telehealth for climate change.

**Results:**

Telehealth has demonstrated success in providing effective healthcare services through teleconsulting and remote monitoring. It has also shown to help reduce carbon dioxide equivalent gas emissions due to reduced travel since it required fewer in-person visits. At the same time, large energy consumption for complex devices and artificial intelligence (AI), Internet of Things (IoT) based data processing applications, e-waste generation from device lifecycle, the potentially negative impact on the quality of care provided with resultant increase in health system encounters undermine the benefits of telehealth. Equity, privacy and security issues are other challenges that need to be considered.

**Conclusions:**

It is possible to integrate telehealth as an integral component of climate change mitigation efforts through a systems thinking approach. The services should be resilient to climate change events, and support mitigation efforts through promotion, prevention, monitoring, management and governance activities. More research is needed to substantiate and investigate this model of healthcare.

## 1. Introduction

Climate change refers to the long term shifts in planet’s temperature and weather patterns [ 1 ]. Over the past 200 years human actions including deforestation, generation of power by burning fossil fuels, overutilization of natural resources, activities that release Greenhouse gases(GHGs) have been the main drivers of climate change [ 2 ]. According to the Intergovernmental Panel on Climate Change (IPCC) report, nearly 3.3 to 3.6 billion people already live in areas highly susceptible to climate change effects [ 2 ]. Air, water, food, weather and every aspect of human life is affected by climate change and its impact on human health and wellness is increasing at an alarming rate [ 3 ]. The world is experiencing a challenge in climate change-induced global disease burden with an increasing incidence of climate-related food-borne, water-borne diseases and chronic disease occurrences [ 3 ]. Respiratory and heart diseases are linked with high temperatures and poor air quality [ 4 ]. Increases in extreme heat events have been reported to result in human mortality and morbidity due to cardiovascular disease. Exposure pathways of carcinogens are affected by climate change events and increase the risk of some cancers [ 5 ]. People living with diabetes are proven to be affected by extreme weather conditions [ 6 ]. The rising burden of mental health is another major global concern. Some of the reported mental health consequences associated with climate change are extreme stress, anxiety, depression, post-traumatic stress disorder, hospital admissions, emergency room visits for mental disorders and suicide [ 7 , 8 ]. There are other climate-sensitive health outcomes like mortality and morbidity due to extreme heat and cold, rise in injuries, higher rates of pre-term birth, still-birth, low-birth weight, adverse child health, and decreased healthcare access that are impacted by hazardous climate change events [ 9 ]. Due to changing climate ecosystems, animals, birds, and insects are facing forced displacement and moving into close human proximity leading to more spread of infections, diseases and increased deaths [ 10 ]. Emerging and re-emerging infectious diseases are becoming a global health problem that require attention [ 11 ].

Telehealth describes various forms of remote consultation that can encompass a range of digital tools, including both video and telephone consultations and digital monitoring devices [ 12 ]. Telehealth services can be provided Synchronously / real-time and Asynchronously /store and forward methods. Virtual health is a term increasingly used in place of or in addition to telemedicine but covers the same concept. There is evidence to suggest that video-supported telehealth is rated as more effective than telephone consultations by patients [ 13 ]. Telehealth has been adopted by many healthcare specialties [ 14 ] with benefits including reduced travel time/cost, increased patient engagement, reduced missed appointments, and improves equity of access to healthcare. It has been viewed satisfactorily by patients [ 13 ] with benefits including less travel, less time away from home and work, and lower costs [ 15 ]. Telehealth services have received increased attention during the COVID-19 pandemic and proven they can support virtual care delivery, including the transition from in-person care to virtual service delivery [ 16 ]. It has also been posited that telehealth can actively reduce GHG emissions and other impacts of climate change through less travel for consults and other appointments [ 17 , 18 ]. With the growing number of elderly citizens (demographic transition), and the growing burden of chronic diseases (epidemiologic transition telemedicine can be a viable solution to provide healthcare services. However, what is lacking is overall guidance for how telehealth can play a sustained role in supporting health service delivery while also helping to manage and mitigate the impacts of climate change.

The severity of climatic risks and their impact on health will be different for everyone. It depends on their exposure to climate change effects, their sensitivity to weather events, the environment, community, their preparedness (safety checks and ready to address the risks) and resilience (avoid, withstand and adapt to change). This is true for the individual as well as the entire health ecosystem. Health systems represent overall health as the intersection of many different system components, including human health, environmental health, and animal health [ 19 ]. The basic tenet of systems thinking is that we must strive to understand the relationships between all system components to enable overall system efficiency and prosperity. While we could optimally configure one part of a system, that could lead to adverse impacts or other unintended consequences in other system components [ 20 , 21 ]. To that end, we must study climate change from the perspective of all aspects of a health system, including digital health tools and approaches such as telehealth, and from the sub-system level of all aspects of digital health tools including hardware, software, data, people/processes and networks. Hence the impact of telehealth on climate change can be both external with the wider system and internal with its’ own sub-systems.

## 2. Scope of the Position Paper

As the International Medical Informatics Association (IMIA) Telehealth Working Group (TWG), our interest is in furthering the science around telehealth design and implementation to promote its potential to improve global health outcomes. We advocate for a systems-based approach to telehealth that considers multiple dimensions of health systems, including technical, financial, human resource, policy and informatics dimensions. Our WG has previously developed a systems-based framework for telehealth to support critical responses such as pandemic management [ 16 ] as well as suggesting how telehealth can support interdisciplinary One Health initiatives that integrate human, animal, and environmental ecosystems [ 21 ]. This paper builds on our previous work by exploring how telehealth could make a sustainable impact on climate change. We first review the evidence on telehealth initiatives, focusing on the progress and challenges faced in their implementation across our global member countries. We then provide a synthesis of these global initiatives and develop a systems-based framework for how telehealth can support the management and mitigation of climate change.

## 3. Methods

The current paper is based on a narrative review conducted on the topic of telehealth and climate change. We examined the following questions “What is the role of telehealth on climate change? How can telehealth help in mitigating climate change risks? What are the barriers and challenges in adopting telehealth for climate change?” Articles published in the last five years were retrieved from Pubmed/MEDLINE database using the following search terms:

**Table:** 

Query
(((“Telehealth” [All Fields]) AND (“Climate Change” [All Fields])); ((“Telehealth” [MeSH Terms]) AND (“Climate Change” [MeSH Terms])); ((“Telemedicine” [All Fields]) AND (“Climate Change” [All Fields])); ((“Telemedicine” [MeSH Terms]) AND (“Climate Change” [MeSH Terms])); ((“Telehealth” [All Fields] ) AND ((“climate change” [All Fields]) AND ((“adaptation” [All Fields]) OR (“mitigation” [All Fields])) ) ); ((“Telehealth” [ All Fields]) AND ((“carbon”[All Fields]) AND ((“emission”[All Fields]) OR (“footprint”[All Fields]))))

The inclusion and exclusion criteria were as follows:

- All peer-reviewed published articles published in English from January 1, 2020, to Dec 31, 2024;
- Letters, viewpoints and editorials were not included;
- Both primary and secondary data analyses were considered;
- Abstract-only articles were not included.

Titles and abstracts were scanned to remove articles that were not related to the use of telehealth and climate change. We included articles related to application of digital technology for climate change. A total of 86 studies were identified from the search. After reviewing the articles, we determined that 42 studies ( Table 1 ) were relevant both to telehealth and climate change.

**Table 1:** Count of the included studies by year of publication.

Year of Publication	Count
2024	11
2023	8
2022	9
2021	9
2020	5

The full text of the 42 articles was further analyzed and based on our objectives, we identified eleven relevant articles ( Table 2 ) for the development of the position paper. A narrative summary was created of the key objective, approach, and findings from each included article. This was followed by an online modified Delphi method among the TWG members. Based on the narrative summary, questions were posed to the TWG members regarding the applicability of telehealth for climate change management, the positive and negative aspects, sustainable strategy and future opportunities. The TWG members also suggested some additional papers relevant to the topic that were included in the findings. The analysis and perspectives from the TWG members were collated to develop a set of themes for this evidence-based position paper on the role of telehealth in addressing climate change.

**Table 2:** Articles finalized to develop themes for the position paper.

Authors	**Year**	**Study Type**	**Methods**	**Findings**
Braithwaite et al. [ 33 ]	2024	Systematic Review	Eight databases and authoritative reports were searched from inception dates to November 2023.	Reductions in carbon emissions resulting from teleconferencing or telemedicine at the micro and meso levels.
Fawad et al. [ 34 ]	2024	Theoretical Approach	A theoretical approach ‘climate responsive telemedicine’ incorporating climate resilience to telehealth services was stated	It is proposed that vulnerability of telehealth services due to climate change events can be addressed by developing infrastructure that can withstand extreme weather events, providing climate change sensitive health care services and building resilient telehealth solutions by integrating solar-powered devices and satellite communications.
Van der Zee [ 25 ]	2024	Systematic Review	Carbon emissions were evaluated based on four categories, including patient travel, and streamlined life cycle assessment (LCA) for assessing included variables relevant to telemedicine	33 articles were included for analysis. The median round trip travel distance for each patient was 131 km. By reducing the travel distance of the patients through telehealth services there is a significant reduction in emissions.
Das & Chandra [ 35 ]	2023	Narrative Review	Summarization of the articles reviewed	AI has challenges in its adoption in the healthcare sector due to financial and operational constraints. AI's carbon footprint is smaller than the overall GHG emission rates in healthcare. Incorporating sustainable goals in AI systems can lead to broader adoption
Thiel et al. [ 18 ]	2023	Retrospective analysis	Environmental Life Cycle Assessment (LCA) to quantify the environmental emissions associated with a virtual and an in-person clinical visit across specialty areas was retrospectively analyzed.	A significant reduction in the GHG emissions by nearly 36% was observed
Marcillo et al. [ 22 ]	2022	Retrospective Study	The average distance travelled by the customers of an insurance company from their home to doctors’ surgery was used to calculate the average distance covered nationally.	The study demonstrated how digital appointments, and digital reports reduce CO2e emissions by reducing the need for patient travel.
Mota et al. [ 32 ]	2022	Qualitative study based on literature review	Agricultural, and environmental surveillance practices of the indigenous community were analyzed	Telehealth communication can be used to transfer these knowledge and practices to the urban community for mitigating climate change
Sijm-Eeken et al. [ 40 ]	2022	Theoretical framework development	Case study, Literature review	A framework combining the concepts from healthcare information technology and environmental sciences was developed that can be used to structure green medical informatics solutions for different healthcare settings. The three components of the framework are monitoring and measuring environmental impact of healthcare, creating awareness and interventions to reduce environmental impacts
Basu et al. [ 16 ]	2021	Online Delphi method	Inputs and perceptions on telehealth use in response to the COVID-19 pandemic by the Telehealth Working Group (WG) of the International Medical Informatics Association (IMIA) was analyzed and mapped with WHO model of health system	A systems-based framework providing guidance for telehealth implementation as part of health systems in response to a global pandemic such as COVID-19 for put forth
Purohit et al. [ 23 ]	2021	Systematic review	Articles related to the carbon footprint of telemedicine were retrieved from the databases MEDLINE, Embase and Scopus.	The total CO2e reductions were divided by the number of consultations to calculate emissions reduction per consultation. Travel distance saved was compared with the carbon footprint reduction per consultation, and linear regressions were used to quantify the strength of this correlation. Carbon footprint savings were highly dependent on the inter-related factors of medical specialty, geography and time.
Wang et al. [ 24 ]	2021	Retrospective cross-sectional analysis	GHG emissions associated with pre-and post- implementation of a novel telehealth preoperative evaluation process was performed on 298 American Society of Anesthesiologists Class I-III patients undergoing elective spine surgery	The study demonstrated that implementing a telehealth preoperative evaluation with standardized pre-operative testing guidelines resulted in reduction in carbon emissions at the rate of 8.09 kg CO2 e (9.6%) per patient. This was due to fewer in-person clinic evaluations. With strict adherence to guidelines, further reduction in emission is expected.

## 4. Results

Table 2 (next page) shows the year, study type, methods, and findings from the 11 articles included in our study. We then present the three themes we identified on the role of telehealth for addressing climate change.

The following three themes were identified from our analysis.

### 4.1. Theme 1: Effectiveness of Telehealth in Addressing Climate Change

Evidence states that telehealth can help mitigate climate change effects by reducing the carbon footprint, lowering energy consumption in healthcare facilities and minimizing waste generation. There are several studies that have proven the effectiveness of telehealth services in lowering the carbon footprint. Thiel *et al.* [ 18 ] evaluated the environmental emissions associated with in-person and virtual clinic visits using a Life Cycle Assessment (LCA) with retrospective clinical data from Stanford Health Care (SHC) in 2019–2021. The LCA was conducted in 4 steps, according to ISO 14040 standards: (1) goal and scope definition, (2) life cycle inventory (LCI), (3) impact assessment, and (4) interpretation and analyses. In addition to this, the energy sources in the examining room including heating, ventilation, air conditioning, the supplies used, and the waste collected were considered. The travelling distance was calculated based on the average distance between the zip code of the patients’ home and the doctor‘s clinic. The GHG emissions were found to be reduced by 36% between 2019 and 2021. Specific specialties like psychiatry and cancer had more virtual visits and hence greater GHG reductions than others.

Marcillo *et al.* [ 22 ] evaluated the reduction in carbon dioxide equivalent (CO2e) emissions due to the use of telemedicine by retrospectively analyzing the environmental impact of the digital health activity. By measuring the average distance between the patient‘s home and the clinic and considering the various modes of transport, the net CO2e emissions differencewas calculated. The study proved that using digital appointments and downloading digital reports resulted in a total of 6,655 tons of CO2e emissions decrease. Purohit *et al.* [ 23 ] calculated the emissions reduction per consultation through a systematic review of the literature retrieved from MEDLINE, Embase and Scopus. The 14 studies included in this review unanimously reported that the reduction in the carbon footprint in comparison to face-to-face interactions was due to travel-associated savings. It was theoretically calculated to range from 0.70 to 372 kg CO2e per consultation but were highly context specific. Wang et al [ 24 ] through a retrospective cross-sectional analysis of pre- and post-operative evaluations of 298 patients undergoing elective spine surgery demonstrated an effective 8.09 kg CO2e emission per patient. In another study, Van der zee [ 25 ] demonstrated the reduction in carbon footprint due to reduced travel through a systematic review. Implementing telehealth services resulted in a net reduction of CO2e emissions. A decrease in physical patient visits to the clinics also reduces traffic congestion and requires less physical hospital infrastructure like rooms, lights, air conditioners, cooling fans, etc. This leads to a further reduction in energy consumption and emissions. Similarly, such reductions in carbon emissions and air pollution savings have been evidenced in telehealth visits for cardiology [ 26 ], orthopedic [ 27 ] and trauma surgery, and pediatric care [ 28 ].

There are a few earlier studies that have documented the multiple health impacts of climate change. The most common are (1) infectious diseases, (2) mortality and (3) respiratory, cardiovascular or neurological outcomes [ 29 ]. By using virtual visits, the energy consumption saved through Telehealth can allow for more targeted interventions to be rolled out and preventive measures can be put in place. People in remote and rural areas where healthcare services are difficult to access are further affected by natural disasters and climate change events. After disasters, travelling to the affected area to provide healthcare services may be a challenge. In addition, access to healthcare facilities in these areas may be affected due to a breakdown in power, network inaccessibility, shortage of essential supplies, resulting in a lack of access to key elements of patient care such as patient records. Many individuals may be affected by mental health issues due to anxiety, evacuation from home, loss of lives and property and financial breakdown and may require counselling and treatment, which may be long term [ 30 ]. Gogia *et al.* [ 30 ] have highlighted their experience of providing tele-mental health services after the tsunami. Specifically mental health support for the victims was provided near their place of residence. Finally, but most significantly, indigenous peoples may respond to natural disasters and climate changes events in different ways requiring different types of holistic support [ 31 ]. In a study by Mota *et al.* [ 32 ] telehealth has been demonstrated as a viable tool to promote and advocate traditional climate change resilient practices adopted by indigenous communities.

### 4.2. Theme 2: Challenges of Telehealth in Addressing Climate Change

While positive effects of the implementation of telehealth initiatives have been evidenced in the form of reduction of CO2e emissions, there are potential disadvantages of using telehealth for addressing climate change. With recent advancements in digital technology, the distinction between telehealth and digital innovation domains begins to blur [ 33 ]. Potential areas of AI application in telehealth are remote monitoring and management, clinical assessment and evaluation, tele-diagnosis and virtual agents. As the complexity grows, more and larger data centers are required to operate telehealth platforms and to process the increasing volume of big data. These consume significant amounts of energy and contribute to greenhouse gas emissions unless they use renewal and low emission energy sources [ 34 ].What is more recognized is that the increased reliance on electronic devices for telehealth can lead to more electronic waste when devices become outdated, posing disposal challenges [ 35 ]. As a part of providing infrastructure to telehealth, there may be contamination of natural environments and subsequent damage to biodiversity in the geographical areas from mining for rare metals leading to significant health risks [ 36 ]. The inherent challenges due to telehealth applications in terms of internet connectivity, remote physician unavailability, and data quality issues are also to be considered. Though use of AI applications can help to address these challenges, there are implicit social, ethical and privacy challenges associated with them. If telehealth is used inappropriately, it might delay necessary in-person examinations or procedures, potentially leading to worse health outcomes and increased hospital visits that further contribute to detrimental environmental impacts. One of the main challenges for telehealth in this regard is equity, as the populations that may benefit the most from telehealth solutions for climate change impacts may not necessarily have access to them. Furthermore, not everyone has equal access to reliable internet and technology, which can exacerbate healthcare disparities and limit the benefits of telehealth in certain populations.

### 4.3. Theme 3: Sustainable Strategies for Implementing Telehealth for Climate Change Mitigation

To implement telehealth for climate change it is important to develop strategies that can help in maximizing its benefits and reduce adverse effects like CO2e emissions. Having considered the challenges for telehealth and climate change, it is interesting that by changing the way in which the underlying components of telehealth are used, telehealth can have a significant positive impact on climate change. Financial levers to reduce emissions from excess procurement, reducing physical waste, energy grid emissions, infrastructure efficiency, and reducing supply chain emissions have been highlighted as some of the strategies that can be adopted to reduce the impact of healthcare on climate change [ 37 ]. The same can be done for telehealth implementation. The devices and technologies utilized should focus on energy conservation, for example with the use of cloud services, solar energy for operating devices, use of low energy-consuming monitors, servers, and network [ 38 ]. It should be possible to utilize these in areas where resources are low. From the design stage to the final implementation and use, the entire telehealth system, including the equipment, devices, and network, should be evaluated for their environmental impact. This evaluation should include life cycle assessment (LCA) to quantify the carbon footprint of devices and infrastructure used for telehealth thus enabling them to prioritize the use of renewable energy solutions. Continuous monitoring and maintenance of the devices, regular upgrades of the hardware to extend the lifetime of the devices, promoting software updates and having a robust e-waste management system go a long way in saving energy and reducing emissions. Recycling old devices to retrieve reusable parts, sharing of devices and computing resources help in reducing e-waste. Increasingly, healthcare organizations are looking towards renewable and efficient energy solutions, intelligent climate cooling and control technologies. AI and ML based models are being developed that deliver more energy-efficient ways of working including reusing the heat produced, to use sustainable energy sources [ 39 ]. For example, AI algorithms can optimize energy consumption of telehealth systems by predicting peak usage times and adjusting the energy use accordingly. This will further reduce the environmental impact.

To reduce unnecessary in-person visits by the patients a systematic evaluation of the needs of the patients and the treatment protocols should be undertaken before promoting telehealth consultations. Every stakeholder should be educated regarding GHG emissions and the ways to reduce them, e-waste disposal and responsible purchasing practices. Telehealth is increasingly being considered as a sustainable strategy for addressing One Health (OH). OH, is a concept that integrates animal, human and ecosystem health that involves multiple stakeholders. Interpersonal collaboration between various stakeholders should be emphasized for data interoperability and interchange. For instance, in regions where climate change has led to the displacement of wildlife and increased the human-animal interactions, telehealth systems can be used to monitor and manage zoonotic diseases. Wearable devices on animals are a new frontier, which can be further utilized for this. By integrating wearable devices on animals, environmental sensors and human health records, telehealth can provide early warnings of potential outbreaks. This can facilitate coordinated responses between human and animal health providers. This in turn will improve the health outcomes as well as contribute to the climate resilience of ecosystems and communities in the face of climate change. Environmental impact of healthcare should be monitored and measured. Awareness and interventions to reduce environmental impacts of healthcare should be part of the climate change mitigation strategies [ 40 ].

## 5. Discussion

As outlined in the earlier sections, one of the main factors contributing to CO2e emissions in healthcare is the travel taken by the patients for face-to-face consultations, health checkups and the travel by the staff, health workers to visit the patients at home and at remote locations. All in-person consultations result in the usage of infrastructure and resources that consume energy. Telehealth offers a way to reduce this reliance on extensive hospital infrastructure by minimizing travel. The COVID pandemic demonstrated that telehealth consultations are well received and efficient in handling doctor patient interactions in addition to reducing the need for patients to travel to healthcare organizations. A prime example is the implementation of tele-ECG centers. By centralizing cardiologists at a main hospital, these specialists can efficiently serve multiple locations remotely. This eliminates the need for each individual hospital to maintain the resources and infrastructure necessary to support on-site cardiologists for ECG interpretation. Lack of access to specialized care may be a contributing factor for migration from remote areas to urban centers. Telehealth can help mitigate this by providing remote consultations and specialized medical services, reducing the need for relocation. This, in turn, alleviates environmental pressure on cities and lessens the strain on urban ecosystems caused by overpopulation. Telehealth facilitates the early detection of health trends within underprivileged communities that might otherwise go underreported, such as the rise in heat-related illnesses, respiratory diseases linked to air pollution, and the spread of infectious diseases driven by climate change. By collecting and analyzing these data, telehealth can provide valuable insights to authorities, supporting evidence-based policy decisions to mitigate environmental and public health risks. Telehealth can be used for monitoring chronic diseases patients remotely to combat the major pitfall of non-adherence of medication and improve outcomes through frequent interactions with care providers. It facilitates the early detection of health trends within underprivileged communities that might otherwise go underreported, such as the rise in heat-related illnesses, respiratory diseases linked to air pollution, and the spread of infectious diseases driven by climate change. By collecting and analyzing these data, telehealth can provide valuable insights to authorities, supporting evidence-based policy decisions to mitigate environmental and public health risks. Telehealth is also increasingly promoted as a core component of One Health and as a first point of contact for monitoring emerging outbreaks. The use of technology such as sensors and AI with telehealth can help address the challenges of telehealth implementation. Using telehealth also comes with challenges and limitations, such as (but not limited to) the lack of training for familiarization with technology, issues of limited access to broadband, especially in remote areas and under-resourced settings, the lack of legal and ethical clarity regarding accountability, data security and privacy issues, a lack of standardized e-waste disposal frameworks, and the excess consumption of computing resources, in particular if the amount of data processing is huge. These challenges can act as roadblocks in the uptake of telehealth as an integral part of climate change mitigation.

## 6. Conclusion: TWG Position on Telehealth for Climate Change

We propose that telehealth be integrated as a core part of addressing climate change mitigation. Telehealth can be used for monitoring health outcomes, creating awareness and education, monitoring and providing telecare for many specialties including mental health and chronic diseases. We advocate for increased design of telehealth systems to support climate-sensitive health outcomes. Point-of-care digital devices, AI-based algorithms, cloud services, wireless sensors and IOT can be integrated with Telehealth tools to enable the design of telehealth tools that support management and mitigation of climate change impacts.

Based on the results of this paper we propose a systems framework that can help in using Telehealth to address climate change issues ( Figure 1 ).

**Figure 1:**
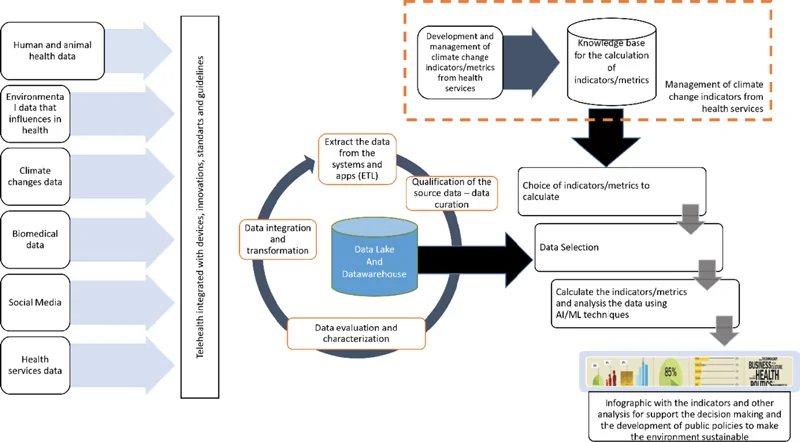
. Systems Framework on Telehealth for addressing climate change issues.

According to the proposed approach, the relevant data on human and animal health related to climate change is collected through telehealth applications, processed, integrated with data collected from other sources, such as environmental data (climate, temperature, humidity, etc.), climate change data (storms, floods, extreme temperatures, etc.), social media, biomedical data and data from health services responsible for climate change. This data is stored in data lakes and data warehouses and then used to calculate climate change indicators for health services and stored in a knowledge base. Machine Learning and generative AI techniques can be used to analyze the indicators and the relationship between them. After calculating the indicators and analyzing them, it is possible to visualize the result in the form of dashboards, infographics and maps in real-time for prediction, prevention, treatment and monitoring of health outcomes that can be implemented through telehealth and remote monitoring. The indicators can be used in decision-making and the development of public policies related to health and climate change.

In summary, telehealth could provide necessary coordination between health (human and animal health) and climate change systems. While telehealth offers potential for mitigating impacts of climate change, to date there is limited research on the impact of adopting telehealth on climate change other than a few studies showing it could lead to a reduction in GHG emissions. There is great opportunity in the potential to adopt telehealth tools as part of sustainable climate change management. Telehealth could help with both the mitigation of climate change impacts (i.e., reducing GHG emissions) and with health management of impacts of climate change. However, more research is needed on the integration of telehealth and climate change to gain insight on how to make healthcare services more resilient and climate sensitive. This paper reviewed the opportunities and challenges of using telehealth as part of climate change sustainability. We also provided a systems framework for how telehealth could help address climate change issues.
